# Impact of Poor Oral Health Status on Swallowing Function Improvement in Older Dysphagic Patients

**DOI:** 10.7759/cureus.51249

**Published:** 2023-12-28

**Authors:** Akio Shimizu, Tomohisa Ohno, Ichiro Fujishima, Jun Kayashita, Ryo Momosaki, Shinta Nishioka, Hidetaka Wakabayashi

**Affiliations:** 1 Department of Food and Health Science, Faculty of Health and Human Development, The University of Nagano, Nagano, JPN; 2 Department of Dentistry, Hamamatsu City Rehabilitation Hospital, Hamamatsu, JPN; 3 Department of Rehabilitation Medicine, Hamamatsu City Rehabilitation Hospital, Hamamatsu, JPN; 4 Department of Health Sciences, Faculty of Human Culture and Science, Prefectural University of Hiroshima, Hiroshima, JPN; 5 Department of Rehabilitation Medicine, Mie University Graduate School of Medicine, Tsu, JPN; 6 Department of Clinical Nutrition and Food Service, Nagasaki Rehabilitation Hospital, Nagasaki, JPN; 7 Department of Rehabilitation Medicine, Tokyo Women’s Medical University Hospital, Shinjuku-ku, JPN

**Keywords:** sarcopenic dysphagia, intervention, older population, oral health, swallowing difficulty

## Abstract

Background

This study aimed to explore the relationship between poor oral health status and improvement in swallowing function in older patients with dysphagia across various clinical settings, including acute and post-acute care environments.

Methods

This retrospective cohort study encompassed patients aged 65 years and older with dysphagia. Oral health status was assessed using the oral health assessment tool (OHAT) or the revised oral assessment guide (ROAG). In this study, an OHAT score of ≥3 or an ROAG score of ≥13 indicated poor oral health status. The primary outcome measured was the change in the food intake level scale (FILS) score, which reflects swallowing function, during the observation period. The association between changes in FILS score and poor oral health status was analyzed using a multivariable linear regression model.

Results

The study included 361 older patients with dysphagia (mean age 82.7 ± 7.7 years; 47.3% male), of whom 82.5% had poor oral health. A negative association was found between poor oral health status and improvement in FILS score at the endpoint (partial regression coefficient: -0.523, 95% confidence interval: -0.99 to -0.06, P=0.026).

Conclusions

Our findings underscore the negative impact of poor oral health status on the improvement of swallowing function and emphasize the importance of oral health interventions for older patients. Further study on oral health interventions' effects on improving swallowing function in older patients with dysphagia is warranted.

## Introduction

Oral health maintenance is crucial for averting adverse outcomes in older patients. Many older adults experience tooth loss, periodontal disease, and other oral health challenges [1]. Furthermore, compromised oral health increases the risk of systemic diseases [2-4]. Moreover, poor oral health status is linked to a heightened risk of aspiration pneumonia [5] and mortality in older patients [6]. These problems can decrease the quality of life for older patients and cause serious health issues. Therefore, it is essential to maintain or improve the oral health status of older patients to prevent these poor prognoses.

Dysphagia, or difficulty swallowing, is common in older patients. The causes of dysphagia include aging, cerebrovascular disease, neuromuscular disease, cognitive dysfunction, cancer, and sarcopenia [7]. Additionally, Alzheimer's disease, which is prevalent in the older population, is often complicated by dysphagia [8]. Dysphagia is a serious problem that increases the risk of aspiration pneumonia, readmission to the hospital, and mortality [7]. Thus, early detection and treatment of dysphagia in older patients are essential to improve their poor prognosis.

Oral health status may influence the improvement of swallowing function in older patients with dysphagia. Several cross-sectional studies have reported an association between poor oral health and lower swallowing function [9,10]. Furthermore, in a single-center cohort study of older patients with stroke, poor oral health status was an inhibitory factor in improving swallowing function [11].

Although associations between poor oral health status and swallowing function in older patients have been reported [9-11], the generalizability of current studies is limited because of their cross-sectional or single-center nature. Therefore, this study aimed to determine these associations using a database that enrolled older patients with dysphagia in multiple settings, including acute care and rehabilitation.

## Materials and methods

Study design and database

This study employed an observational cohort design and utilized the sarcopenic dysphagia database [4,12]. Although a detailed description of this database can be found elsewhere [12], a brief overview is as follows: The database comprises patients aged ≥20 years diagnosed with dysphagia, defined as a condition in patients with a food intake level scale (FILS) [13] score of ≤8 [14-16]. The FILS scores are categorized as follows: 1-3 for various degrees of non-oral intake; 4-6 for degrees of oral food consumption alongside alternative nutrition; 7-8 for solely oral food intake; 9 when there are no dietary restrictions but with medical considerations; and 10 for regular oral food consumption [13]. Additionally, this database includes patients admitted to multiple settings, including acute, rehabilitation, and long-term care wards. The eligibility criteria for this study included patients aged ≥65 years who were registered in the database. Exclusion criteria for this study were patients with missing data on oral health status and swallowing function.

Ethical considerations

The database was established with the approval of the Yokohama City University Hospital Ethics Committee (B190700074). For ethical considerations, participants in this study at each research facility were either informed and consented before participation or were provided with an opt-out procedure, ensuring their right to withdraw from the study at any time.

Patient characteristics

The databases registered various variables, including age, sex, primary disease, Charlson comorbidity index (CCI) [17], body mass index (BMI), presence of sarcopenia defined by the Asian Working Group for Sarcopenia 2019 criteria [18], Barthel index [19], and hospital types. The primary disease in this database is recorded according to the International Classification of Diseases, 10th Revision (ICD-10) code. In this study, the primary diseases were classified into diseases of the circulatory system (I00 to I99), injury, poisoning, and other external causes (S00 to T88), diseases of the respiratory system (J00-J99), and other diseases based on the proportion of ICD-10 codes. Based on the previous studies [4,20], CCI scores were classified as >2 points and ≤2 points. At each facility, sarcopenia was assessed based on decreased muscle mass, along with either decreased handgrip strength or impaired physical function. Each facility used either bioelectrical impedance analysis, dual-energy X-ray absorptiometry, or calf circumference for muscle mass measurement in sarcopenia assessment. Patients with Barthel index scores of ≤75 points were identified as having activities of daily living (ADL) dependence [4]. Hospital types were classified as acute care, rehabilitation, long-term care, and other hospitals.

Oral health status evaluation

Each facility evaluated oral health status using at admission either the oral health assessment tool (OHAT) [21] or the revised oral assessment guide (ROAG) [22]. These assessments were conducted by professionals from dental fields, such as dentists and dental hygienists, as well as those from non-dental fields, including nurses and speech-language pathologists. These tools have been validated in non-dental professionals [21,22]. The OHAT evaluates eight domains of oral health (lips, tongue, gums and tissues, saliva, natural teeth, dentures, oral cleanliness, and dental pain), scoring on a 3-point scale (0 points for a healthy state, 1 point for changes, and 2 points for an unhealthy state), resulting in scores ranging from 0 to 16 points. The ROAG evaluates eight domains (voice, lips, saliva, swallow, mucous membrane, tongue, gums, and teeth/dentures), with scores ranging from 8 to 24. This study considered oral health poor if the OHAT score was three or above [3,4,11] or the ROAG score was 13 or above [4].

Outcome

The primary outcome in this study was the FILS score [13] at the endpoint, which indicated swallowing function at the endpoint. FILS is commonly used in Japan as an indicator of swallowing function. The study also examined whether the association between poor oral health and improvement in swallowing function was consistent across oral health measures.

Statistical analysis

For statistical variables, we presented categorical variables as numbers (percentages), represented continuous variables as mean ± standard deviation, and described ordinal variables as median (interquartile range). We used the chi-square test for categorical variables, applied the t-test for continuous variables, and used the Mann-Whitney U test for ordinal variables. We utilized a multivariate linear regression model to elucidate the relationship between poor oral health status and FILS score at admission and discharge. Further, the association between improved swallowing function at discharge and poor oral health status was analyzed using a multivariate logistic regression model. We identified confounding factors through directed acyclic graphs and drew upon references from previous studies [14,23]. The multivariate analysis accounted for confounding factors including age, sex, primary diseases (diseases of the circulatory system, injury, poisoning, and other external causes, diseases of the respiratory system, and other diseases), CCI >2 points, BMI (kg/m^2^), presence of sarcopenia, ADL dependence (BI ≤75 points), FILS at baseline, and hospital types (acute care or other hospitals). Sub-analyses were conducted to stratify poor oral health status as assessed by OHAT and ROAG and analyze its association with swallowing function at discharge. All analyses were performed using R version 4.2.3 (The R Foundation, Vienna, Austria) and set the statistical significance threshold at P-value < 0.05.

## Results

**Figure 1:**
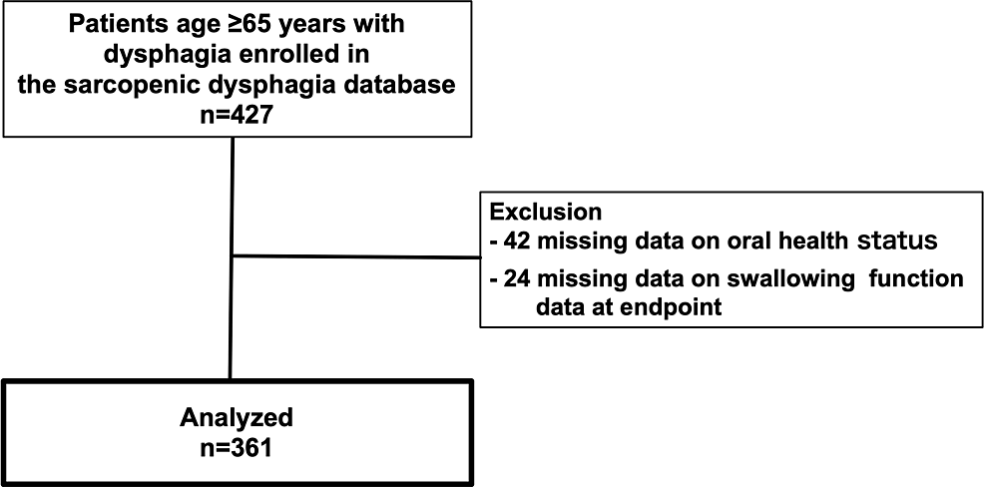
Flowchart of study participants

We adapted the eligibility criteria for 427 older patients aged ≥65 years from the sarcopenic dysphagia database. Of these, 42 had missing data on oral health status at admission, and 24 were excluded from the study because of missing data on the endpoint swallowing function. Ultimately, we analyzed data from 361 older patients with dysphagia (Figure 1).

Table 1 presents the characteristics of the older patients with dysphagia categorized by their oral health status. Of the 361 patients, those with poor oral health status totaled 298 (82.5%), while 63 (17.5%) had normal oral health status. No differences in mean age were observed between the two groups (P=0.967). The group with poor oral health status had a significantly lower FILS score of 7 (2-7) at baseline compared to the group with normal oral health status, which had a FILS score of 7 (7-8) (P<0.001). There was a significant difference in the rate of hospital type between the two groups (P<0.001).

**Table 1:** Baseline patients' characteristics ^a^ADL dependence was defined as a Barthel Index score of ≤75 points. Abbreviations: SD, standard deviation; IQR, interquartile range; CCI, Charlson comorbidity index; BMI, Body mass index; ADL, Activities of daily living; OHAT, oral health assessment tool; ROAG, revised oral assessment guide; FILS, food intake level scale.

	Poor oral health status	Normal oral health status	P value
Number (%)	298 (82.5)	63 (17.5)	
Female sex, n (%)	151 (50.8)	35 (55.6)	0.588
Age, years, mean (SD)	82.2 (7.5)	82.1 (8.7)	0.967
Disease category, n (%)			0.001
Diseases of the circulatory system	101 (33.9)	23 (36.5)	
Injury, poisoning, and other external cause	82 (27.9)	30 (47.6)	
Diseases of the respiratory system	40 (13.4)	6 (9.5)	
Other disease	74 (24.8)	4 (6.3)	
CCI > 2 points, n (%)	116 (38.9)	19 (30.2)	0.245
BMI, kg/m^2^, mean (SD)	20.4 (3.7)	19.8 (4.0)	0.331
Presence of sarcopenia, n (%)	254 (85.2)	57 (90.5)	0.290
ADL dependence^a^, n (%)	280 (94.0)	57 (90.5)	0.480
OHAT score (n = 137), median (IQR)	6 (4–7)	1 (1–2)	<0.001
ROAG score (n = 224), median (IQR)	12 (10– 15)	8 (8–8)	<0.001
FILS score, median (IQR)	7 (2–7)	7 (7–8)	<0.001
Hospital type, n (%)			<0.001
Acute care hospitals	138 (46.3)	9 (14.3)	
Rehabilitation hospitals	125 (41.9)	47 (74.6)	
Long-term care hospitals	33 (11.1)	7 (11.1)	
Other hospitals	2 (0.7)	0 (0.0)	

Table 2 presents the comparison of the variables at the endpoint. While no difference was observed between the two groups during the follow-up period, the poor oral health status group had a significantly lower FILS score of 7 (7-8) compared to the normal oral health status group with a FILS score of 8 (7-9) (P<0.001).

**Table 2:** Comparison of variables at the endpoint Abbreviations: IQR, interquartile range; FILS, Food Intake Level Scale.

	Poor oral health status	Normal oral health status	P value
Length of hospital stay, median (IQR)	52 (25–89)	60 (41–89)	0.123
FILS score, median (IQR)	7 (7–8)	8 (7–9)	<0.001

**Figure 2:**
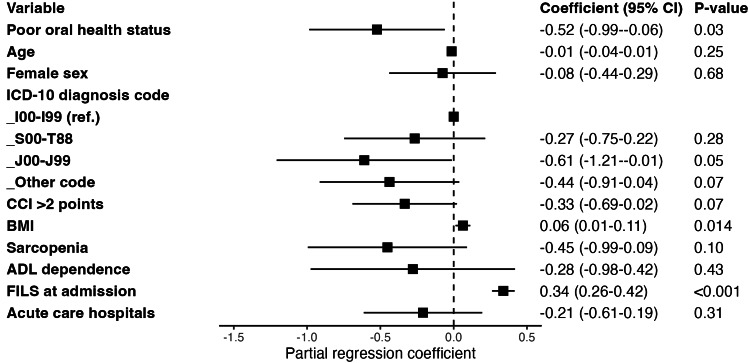
Relationship between poor oral health status and improvement of swallowing function Primary diseases were classified into diseases of the circulatory system (I00 to I99); injury, poisoning, and other external causes (S00 to T88); diseases of the respiratory system (J00 to J99); and other diseases based on the proportion of ICD-10 codes. Abbreviations: 95% CI 95% confidence interval; ICD-10, International Classification of Diseases, 10th Revision; CCI, Charlson comorbidity index; BMI, Body mass index; ADL, Activities of daily living; FILS, food intake level scale.

Figure 2 shows the results of a multivariate linear regression model on oral health status and FILS scores at an endpoint. Poor oral health status was inversely associated with FILS scores at the endpoint (partial regression coefficient: -0.52, 95% confidence interval: -0.99 to -0.06, P=0.03). Additionally, BMI was positively associated with FILS score (partial regression coefficient: 0.34, 95% confidence interval: 0.26-0.42, P<0.001).

**Figure 3:**
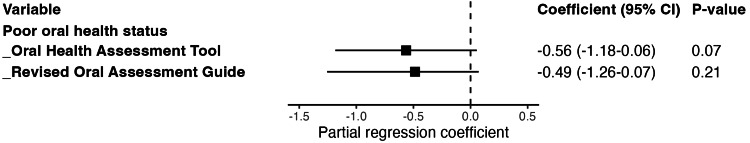
Relationship between oral health status for each oral health assessment tool and improvement of swallowing function A multivariate model was adjusted for age, sex, primary disease, Charlson comorbidity index >2 points, body mass index, sarcopenia, activities of daily living dependence, food intake level scale at admission, acute care, or other hospitals. Abbreviations: 95% CI 95% confidence interval.

Figure 3 shows the results of a multivariate linear regression model for each OHAT. Poor oral health status as assessed by OHAT (partial regression coefficient: -0.56, 95% confidence interval: -1.18 to 0.06, P=0.07) and ROAG (partial regression coefficient: -0.49, 95% confidence interval: -1.26 to 0.07, P=0.21), and both tended to be negatively associated with FILS score at the endpoint.

## Discussion

This study utilized a database of older patients with dysphagia, encompassing various primary diseases and settings, to evaluate the potential impact of poor oral health status on the improvement of swallowing function. Our analysis of 361 older patients with dysphagia revealed that poor oral health status negatively affected the improvement of swallowing function. However, results varied across different OHATs.

In older patients with dysphagia, poor oral health status was identified as a detrimental factor to the improvement of swallowing function. In our study, patients with poor oral health status at admission had lower swallowing function than those with normal oral health status. Poor oral health status includes loss of natural teeth, poor fit of dentures, and reduced salivary flow. Thus, poor oral health status may adversely affect swallowing function by limiting the foods that can be ingested. On the other hand, poor oral health status may be recognized because of poor swallowing function. Lower swallowing function is associated with xerostomia [9]. For these reasons, poor oral health status may have been associated with lower swallowing function at admission in this study. The results were similar to previous studies showing a cross-sectional association between poor oral health status and poor swallowing function [9]. Further, previous studies have demonstrated a correlation between poor oral health and reduced progress in swallowing function in patients with stroke and aspiration pneumonia [24-26]. This study, focusing on older patients with various diseases, echoed these findings. Although disease is a factor that influences improvement in swallowing function, the association between poor oral health status and improvement in swallowing function was consistent even after accounting for the influence of disease in the multivariate analysis. Poor oral health can lead to adverse outcomes, such as inflammation and aspiration pneumonia [5], which can obstruct functional recovery, including swallowing rehabilitation, in patients with dysphagia. On the other hand, compromised oral health might hinder modifications in food texture due to impaired masticatory function, which is crucial for changing food texture. Tooth loss and the use of dentures, integral aspects of oral health, affect chewing ability. Additionally, a previous study has indicated that consuming chewy foods enhances masticatory function [27]. Additionally, decreased salivation is often thought to be associated with dysphagia [24]. It has been suggested that xerostomia may adversely affect the swallowing process and impair bolus formation and transfer [24]. Thus, impaired oral health could restrict food texture modification, further hampering the improvement of swallowing function.

The influence of poor oral health on swallowing function was inconsistent across the employed OHATs. A prior systematic review affirmed the validity of OHAT and ROAG in assessing oral health in older patients [28]. Despite this, a potential limitation in our sub-analysis might be inadequate statistical power. Nevertheless, the 95% confidence interval for poor oral health, as defined by OHAT, suggested a tendency towards a negative impact on swallowing function improvement. The lack of a definitive link between ROAG and swallowing function improvement could be attributed to the cutoff value for poor oral health and the demographic distribution of the study participants. Notably, previous studies have highlighted the negative impact of poor oral health, as measured by ROAG, on clinical outcomes [29,30]. This means that the results of this sub-analysis should be interpreted with caution. Furthermore, different domains of OHAT and ROAG may have influenced the sub-analysis results. OHAT does not include voice or swallow, which are not included in ROAG. These endpoints may have influenced the improvement in swallowing function. However, this study did not adapt two oral hygiene assessment tools for the same patient. Future studies are needed to determine whether OHAT or ROAG is more associated with improved swallowing function.

The strength of this study was the use of a database involving multicentric and patients with disease to determine the association between poor oral health status and improved swallowing function.

The study has several limitations. First, the database lacks detailed records of patients' treatments, which can significantly influence clinical outcomes. Second, it did not incorporate objective evaluations of swallowing function, such as fiberoptic endoscopic evaluation of swallowing or videofluorographic swallowing study. Despite this, numerous studies have used FILS as a clinical evaluation tool for swallowing function [14,16]. Third, this study employed an opt-out procedure to comply with ethical standards. While this approach facilitated the participation of more individuals, it may have introduced potential bias. To avoid potential bias, explaining and obtaining consent for participation in the study in person may have been desirable. Finally, the absence of facility variables in the database prevented analysis using generalized estimating equations to consider cross-facility effects.

## Conclusions

This study demonstrates that poor oral health status hinders the improvement of swallowing function in older patients with dysphagia across various settings. Despite some limitations, enhancing oral health could potentially contribute to better swallowing function. Further studies focusing on oral health interventions and improving swallowing function in older patients with dysphagia are warranted.
